# Behavioral Plasticity in Probing by *Diaphorina citri* (Hemiptera, Liviidae): Ingestion from Phloem Versus Xylem is Influenced by Leaf Age and Surface

**DOI:** 10.1007/s10905-018-9666-0

**Published:** 2018-02-20

**Authors:** Timothy A. Ebert, Elaine A. Backus, Holly J. Shugart, Michael E. Rogers

**Affiliations:** 1 Department of Entomology & Nematology, Citrus Research and Education Center, University of Florida, 700 Experiment Station Rd, Lake Alfred, FL 33850 USA; 2 USDA, Agricultural Research Service, San Joaquin Valley Agricultural Sciences Center, 9611 South Riverbend Avenue, Parlier, CA 93648-9757 USA

**Keywords:** Experimental design, multivariate analysis, discriminant analysis, insect, feeding, electropenetrography, electrical penetration graph, EPG

## Abstract

*Diaphorina citri* is a major pest of citrus because it transmits *Candidatus* Liberibacter asiaticus, a phloem-limited bacterium that putatively causes Huanglongbing (HLB). The disease moves slowly through a tree, and the vector facilitates further within-tree movement via transmission of the pathogen. However, this only happens when *D. citri* stylets contact the phloem, to inoculate bacteria during phloem salivation and acquire bacteria during phloem sap ingestion. Behavioral changes in *D. citri* associated with different plant parts would affect how long it takes to reach phloem and how long the psyllids stays in phloem to ingest, thereby influencing the risk of disease spread. *D. citri* feeding was recorded on the abaxial and adaxial surfaces of mature and immature citrus leaves. Adults in the field can be found on these surfaces at all times of year. On abaxial surface of immature leaves, phloem salivation would occur after 11 h on average, but rarely as soon as 0.56 h. The corresponding values on mature leaves were 16 and 2.7. In general, psyllids spent more time ingesting phloem sap on immature leaves than on mature leaves. Psyllids on abaxial surfaces spent more time ingesting from phloem, though the strength of this effect was less than for immature versus mature leaves. In contrast, xylem ingestion increased on mature leaves compared with young. The biological differences that could produce this outcome are discussed. The results discussed herein are of relevance to further studies on the efficacy of an insecticide to act quickly enough to prevent pathogen transmission.

**Electronic supplementary material:**

The online version of this article (10.1007/s10905-018-9666-0) contains supplementary material, which is available to authorized users.

## Introduction

The Asian citrus psyllid (*Diaphorina citri* Kuwayama: Hemiptera, Laviidae) transmits (acquires, retains, and inoculates) a phloem-limited, unculturable, alpha proteobacterium (*Candidatus* Liberibacter asiaticus [*C*Las]) that is the putative causal agent of Huanglongbing (HLB). Both the pathogen and its vector are highly invasive species presenting global challenges to the citrus industry. In the U.S., the first record of the psyllid in Florida was in 1998, and the disease was first detected in Florida in 2005 (Halbert 2005). The disease reduces fruit quality and causes tree mortality, thereby resulting in extensive economic losses (Hodges et al. 2014). The psyllid was reported from Texas for the first time in 2001 (French et al. 2001), and reported for the first time from California in 2008 (Grafton-Cardwell 2010). The disease was first detected in both Texas and California in 2012 (Kumagai et al. 2013; Kunta et al. 2014b). In 2016, *D. citri* was found for the first time in Africa (Shimwela et al. 2016), and both vector and pathogen are expected to spread to all citrus producing regions worldwide (Narouei-Khandan et al. 2016).

A number of HLB disease management strategies are being developed (Grafton-Cardwell et al. 2013; Hall et al. 2013), with the ones most relevant herein being those strategies focused on vector control using insecticides or host plant resistance against the psyllid. Success of these strategies is not dependent on insect mortality alone, because it may be possible for the target psyllid to transmit the pathogen prior to dying, thus making the insecticide application and/or some types of host plant resistance ineffective for disease management.

For acquisition, it is important to understand how the insecticide influences the insect’s ability to achieve phloem sap ingestion following salivation on infected plants. Naturally, acquisition would only be important in cases where the insect lives long enough to disperse to another tree or part of the same tree and then inoculate a healthy host or to move the pathogen within an infected host in cases where the pathogen is not already ubiquitous within that host (respectively). This latter condition is important in the *C*Las-citrus system, as the pathogen is not uniformly distributed (Kunta et al. 2014a; Li et al. 2009; Louzada et al. 2016). Acquisition of *C*Las is more efficient in older nymphs than any other stage (Xu et al. 1988). However, distribution of nymphs is dependent on oviposition site selection by adult females. *D. citri* adults can be found on all aerial parts of the tree in a citrus grove. However, eggs are only laid close to apical meristems (Huang et al. 1999; Yang et al. 2006). First and second instar nymphs can only survive at this location, although older nymphs can survive on older leaves (Arredondo de Ibarra 2009).

*C*Las is a persistent, circulative pathogen, and therefore is inoculated (after a latent period) during salivation from the vector’s infected salivary glands. To understand inoculation, phloem salivation in healthy plants is most important. Inoculation has been experimentally successful with inoculation access periods (IAP) ranging from 15 min to 7 h (Grafton-Cardwell et al. 2013; Inoue et al. 2009; Pelz-Stelinski et al. 2010). This range is important because there is variability in the time it takes an individual psyllid to reach phloem. Thus, with individual psyllids there is some probability that it will take 15 min or less, though it is likely to take much longer. The IAP will decline as the number of psyllids increases because it is more likely that at least one individual will reach phloem within a short time.

We are especially interested in understanding how insecticides influence the ability of *D. citri* to reach the phloem, thus expanding on earlier work (Serikawa 2011; Serikawa et al. 2012; Serikawa et al. 2013). One question we had was whether psyllid behavior was the same no matter where or when it occurred on the plant. This is an important point if the purpose of an insecticide application is to prevent *C*Las transmission, because one needs to conduct tests on the plant organ where the psyllid is most likely to reach the phloem. If organ age or surface does not matter for transmission of *C*Las, then one could conduct tests wherever it was convenient. Our experimental design also addressed several additional questions: 1) Assuming that there is an effect of adaxial versus abaxial locations and an effect of immature versus mature leaves, which effect is greater? Answering this question will improve experimental methodologies by identifying the approach where the psyllid’s stylets reach phloem as quickly as possible and remain in the phloem for the greatest length of time. This optimal location provides the overall best chance to acquire or inoculate the phloem limited pathogen. 2) Is xylem sap ingestion a natural part of psyllid behavior, or an artifact of stress caused by wiring for EPG and possibly stress from the short starvation period necessary to wire the psyllids? This issue is important because immature tissues are more abundant at some times of year, and it is useful to know whether the probability of phloem contact is the same regardless of when or where the psyllid is probing. 3) How fast must an insecticide work to prevent acquisition and/or inoculation of *C*Las? Understanding this timing is critical in designing bioassays that test insecticide efficacy for vector management as a tool for pathogen management.

While *D. citri* adults can be found on all above-ground parts of the tree, the psyllid generally prefers to settle on the abaxial leaf surface close to the midvein (Arredondo de Ibarra 2009). However, presence does not necessarily equate to probing and ingesting. It is not known whether the psyllid can ingest phloem or xylem from all of these organs. To better understand the probing behavior of *D. citri* we examined the probing behavior of this insect on immature and mature leaves on both adaxial (upper) and abaxial (lower) leaf surfaces.

## Materials and Methods

### Plants

We used citrus resets that were cv. ‘Midsweet’ orange scion on cv. ‘Kuharsky’ rootstock (Southern Citrus Nurseries Inc., Dundee, FL: certified *C*Las free), both for rearing psyllids and as test plants. Plants were pruned and fertilized (variously with Chelated Citrus Nutritional Spray from Southern Agricultural Insecticides Inc. Palmetto, FL, USA, Miracle-Gro All-Purpose Plant Food, Scotts Miracle-Gro, Marysville, OH, USA, or Harrell’s Profertilizer 12–3-8, Harrell’s LLC, Lakeland, FL) consistent with label instructions to promote flush. All plants had flush, even those used to test mature leaves.

**Fig. 1:**
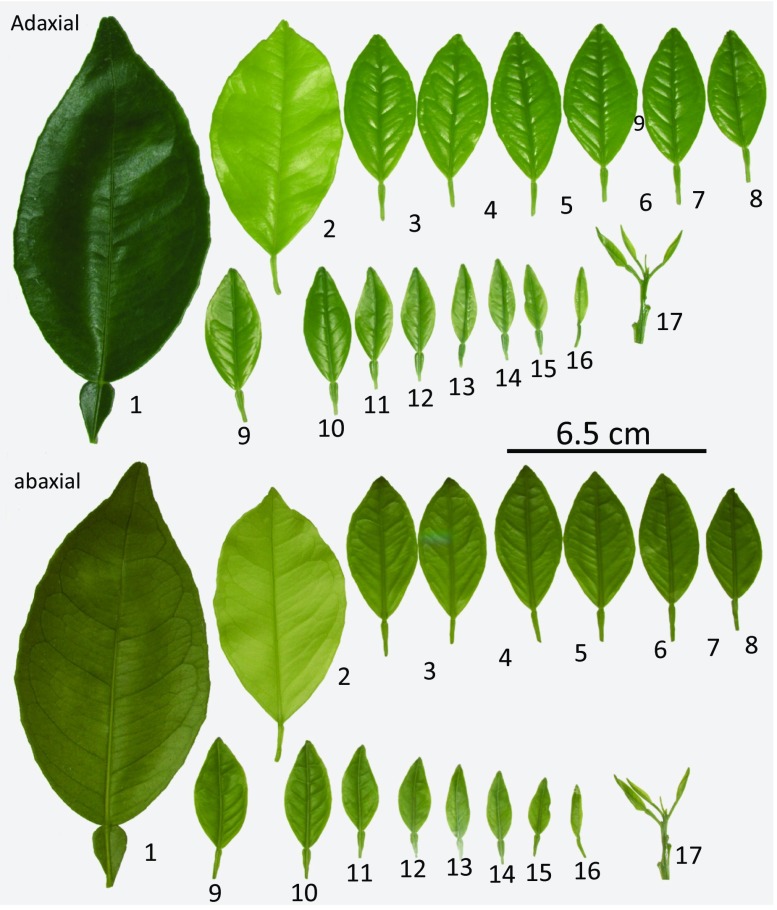
The sequence of leaves showing color differences between a mature leaf (#1), a fully expanded leaf (#2), immature leaves (#3-#16), and the youngest leaves at the apical meristem (#17). In this sequence, leaves #5-#12 would be suitable for testing. Note that the mature leaf was from the same plant as the other leaves, but was not part of the flush

Mature leaves were dark green, fully expanded, and at least one-month old. Immature leaves were light green, not fully expanded, of variable age (four to ten days old). The main criterion for defining leaf status was leaf stickiness. If one rubbed a finger over a mature leaf, the waxy coating made the leaf feel slick. One could apply pressure, and the finger would slide across the surface without causing damage. Such leaves are found close to the apical meristem of new flush (Fig. 1). Immature leaves have a lighter green color but even fully expanded leaves are still light green (Fig. 1). A finger rubbed across the surface will tend to stick (slightly), and with pressure, the leaf will roll and tear. After initial training, immature leaves are easily identified by a light touch, or subtle changes in color. Only undamaged leaves were used in experiments. Under our growing conditions, immature leaves measured 1.0 to 2.5 cm wide and 2 to 4 cm long. Mature leaves measured 4.3 to 6.2 cm wide by 6.4 to 10 cm long. Immature leaves are less than half the size of a mature leaf on these plants.

### Insects

Psyllids were from a laboratory colony that was started in 2006 from field-collected material. This was considered a *C*Las-free colony because *C*Las was first reported in Polk county, Florida, August 24, 2007 (http://www.crec.ifas.ufl.edu/extension/greening/history.shtml). Plants for psyllid rearing were selected from the plants reared under conditions described above. The psyllid colony was contained in fabric cages (Bioquip 1466BV: Bioquip.com, Rancho Dominguez, CA, USA) in the same greenhouse used to grow plants. We did not determine sex or age of the adult psyllids prior to recording.

### Experiment

Adult psyllids were selected arbitrarily from the colony for testing. Plants with flush were arbitrarily selected from the greenhouse from all the plants with flush. The greenhouse had 200 plants, but not all plants flush at the same time. A total of 100 insects were recorded, 37 for the immature abaxial treatment, 19 each for the immature adaxial and mature adaxial treatments, and 25 for the mature abaxial treatment. Insects that jumped or walked off the plant were eliminated from the analysis, explaining the unequal sample sizes.

### Electropenetrography

These methods have been reported before (Ebert and Rogers 2016). A 25.4 *μ*m diameter gold wire (Sigma Cohn Corp., Vernon, NY) was attached to an adult psyllid pronotum using silver glue. A ~30 min starvation period was sufficient time to collect, wire, and set up the experiment. This starvation period is somewhat longer than the average flight duration period where blue-green females would fly an average of 14 min, while brown males would average 0.8 min (Martini et al. 2014).

We used two four-channel AC-DC monitors (Backus and Bennett 2009) that were custom-built by EPG Technologies, Inc. (Gainesville, FL), set to input impedance of 10^9^ and 150 mV DC substrate voltage. Gains used were 160× adjusted amplification (on the control box) and 100× fixed amplification in the head-stage amplifier. Light was provided by overhead fluorescent lights (24:0 L:D) at 26.6 °C.

The equipment was set to record for 24 h because a shorter recording time would not be adequate to identify time to first phloem ingestion for many individuals (see **Results**). Data acquisition used Windaq lite acquisition software through a DI710 A/D converter (both from Dataq.com, Akron, OH) sampling at 100 Hz/channel. Waveforms were manually identified and measured using Windaq Waveform Browser (Dataq.com). We used only the original six psyllid waveforms (NP [non-probing], C [pathway], D [phloem contact], E1 [phloem salivation], E2 [phloem ingestion], and G [xylem ingestion]) (Bonani et al. 2010). Five other waveforms have been described that provide additional biological detail in the non-probing and pathway waveforms (Cen et al. 2012; Pearson et al. 2014; Yang et al. 2011; Youn et al. 2011). These waveforms may be critical in understanding host plant resistance issues. However, we did not use these waveforms because it was not necessary for this experiment. At the end of each recording, a behavioral event occurs that ends prematurely because we stopped the recording. This event was deleted.

### EPG Data Analysis: Programs and Variables

All of our analyses used the Ebert 2.0 analysis program, http://www.crec.ifas.ufl.edu/extension/epg/sas.shtml that runs within the SAS® software environment. We used SAS software release 9.4 M4 running under SAS Enterprise Guide 7.13. There were a total of 85 variables available for the following analyses (see supplement for full list).

The initial analysis used Proc GLIMMIX, which uses Reduced Maximum Likelihood methods for mixed model ANOVA, resulting in improved power of statistical tests (Gbur et al. 2012). Within this procedure, means were separated using the LSD test, with α = 0.05. Data were transformed prior to analysis (typically, log transformation for durations, square root transformation for frequencies). We transformed percentages as log(p/(1-p)) (Warton and Hui 2011), but a few cases had Q-Q plots closer to normally distributed when we used the arcsine square root transformation. See Table S-1 in Supplemental Information for a list of all the variables and the transformations.

### Data Analysis: Transitional Probabilities

Ebert 2.0 also was used to calculate the number of transitional events for each waveform. The arrows in our behavioral kinetogram (see **Results**) were produced by dividing these values by the total number of transitional events in each treatment. The circles in our behavioral kinetogram have an area proportional to the total recording time for each treatment. For both the arrows and the circles, there is only a single, summed value for each treatment. Therefore, statistical analyses are not possible.

### Data Analysis: Univariate ANOVA Analyses

EPG recordings are a temporal sequence of specific behavioral events. We can measure how soon they occur, or how many of them occur after some specific event. We can calculate counts, means, means of means, standard deviations, and a variety of other summary statistics. Our overall goal is to describe how the insect behaves. In this task, it is important to know which behaviors showed significant differences and which ones did not. We suspect that certain behaviors are constrained at the species, genus, or family level. Thus, a lack of significance is not sufficient to decide that a specific variable is unimportant. Despite this acknowledgment, it is typical to only report variables that are significant. Therefore, we have placed the results for all variables in the supplemental material.

### Data Analysis: Multiple Regression Analysis

We performed a regression analysis to explicitly test the effect of age, side, and their interaction. The regression model used in our study required the creation of two binary dummy variables. One dummy variable codes for leaf age, the other for the side of the leaf.

### Data Analysis: Multivariate Analyses

We performed a stepwise discriminant analysis (SAS, Proc StepDisc) to reduce the 85 variables by identifying ones that had the greatest explanatory power and were least correlated with other variables in the model. Using the reduced number of variables, we ran a canonical discriminant analysis (SAS, Proc CanDisc) to visualize the results by plotting the first two canonical correlates. A discriminant analysis (SAS, Proc Discrim) was run to calculate Mahalanobis distances and the classification error rate. We used the nonparametric method with a kernel of 1, 2, or 10. We only report results for kernel of size 1 because that model had the lowest classification error rate.

Histological comparison of sweet orange cultivars: In the time it has taken to complete the EPG portion of this experiment, several manuscripts were published that conducted parallel experimental design to ours. In an effort to place our experiment into context with these other studies, we chose to include a histological comparison of the immature and mature leaves of the sweet orange cultivar used in our study (Midsweet) compared to the cultivar (Valencia) used in another similar study (George et al. 2017). We knew from previous histological studies performed in our lab, that these two cultivars have nearly identical leaf anatomy, are both highly preferred by *D. citri*, and are thus both excellent cultivar choices for EPG studies on baseline behavioral preferences. One important difference between ours and the George et al. (2017) study is the choice of leaf age used for EPG recordings. Leaves equivalent to leaves 9 through 13 in Fig. 1 were used in this study. A mature leaf (Fig. 1, leaf 1) was also examined for comparison, and to provide continuity with previously published work such as George et al. (2017).

Immature and mature sweet orange leaf sections, cultivars Midsweet and Valencia, were excised and processed for histological and microscopic examination. Samples of midvein (3–4 mm) were excised following *D. citri* feeding, and placed in 6% paraformaldehyde in HEPES buffer for fixation. Samples were then processed through a standard tert-butanal-ethanol dehydration series, infiltrated with and embedded in paraffin wax. Wax embedded blocks were then sectioned to a thickness of 8 μm using a rotary microtome (Microm HM355, Waldorf, Germany). Sections were arranged serially on slides and allowed to dry at 42 °C. Sections were de-waxed using 100% xylene, stained using 0.5% aqueous safranin, and counter-stained using 0.01% ethanolic fast green, then cover-slipped using Permount mounting medium (Thermo Fisher, Waltham, MA, USA). Slides were visualized using brightfield settings on an Olympus BX61 compound microscope. Digital images were captured using a 14 megapixel OMAX (Irvine, Ca, USA) model A35140U camera, and ToupViewX (Hangzhou ToupTek Photonics Co., Ltd.; Zhejiang, P.R.China) software.

## Results

### Transitional Probabilities

**Fig. 2:**
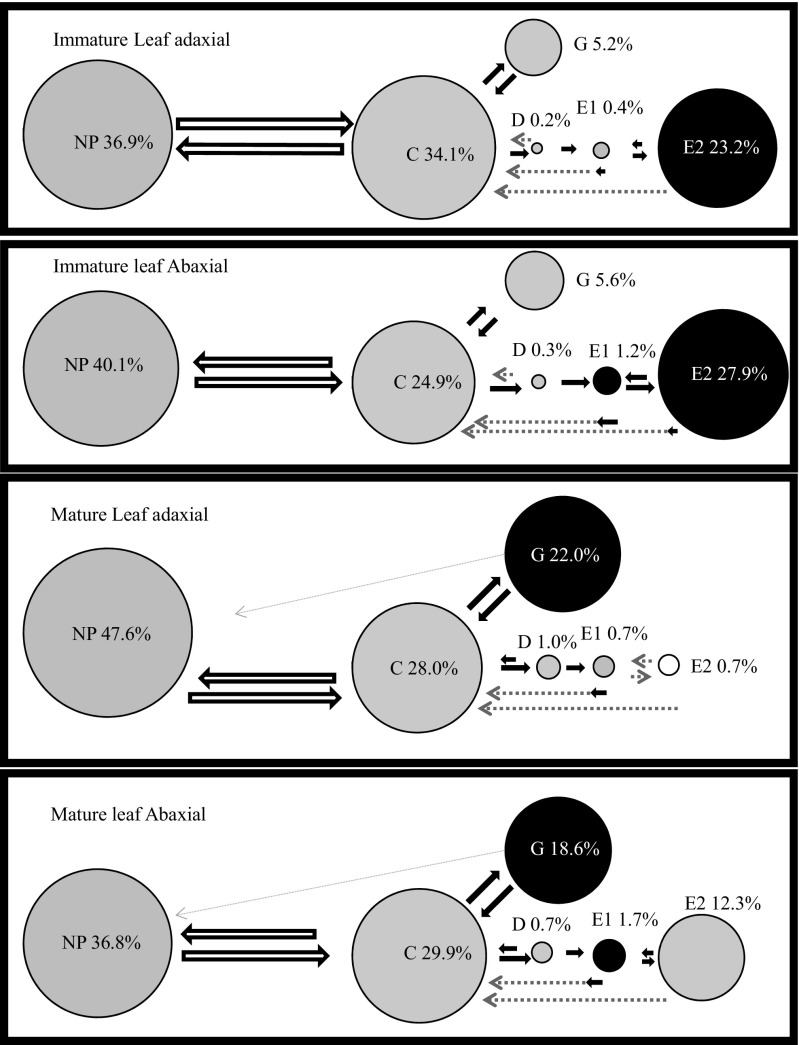
Behavioral kinetogram of *Diaphorina citri* feeding on immature versus mature leaves and the abaxial versus adaxial surface of those leaves. The durations (circle area) of behaviors with different colors are significantly different (Supplement Table). Arrows represent transitions, with arrow size proportional to frequency. Some arrows are broken lines because the proportional arrow size does not make the transition clear

Figure 2 is a behavioral kinetogram where the sizes of the arrows are proportional to the probability of a certain waveform event taking place, given the type of waveform that preceded it. The data summarized are the summed counts from all insects in each treatment. Non-probing always transitions to probing/pathway (C). Of the three possible transitions after pathway (NP, G, or D), returning to non-probing (NP) is the most common. Probing/pathway can transition to xylem ingestion (G), which has a longer duration (larger circle) on older leaves. While xylem ingestion usually transitions back to pathway, only in rare cases will it transition directly back to non-probing. Phloem contact (D) is the third possible transition from pathway, and thereafter the insect can either go on to phloem salivation (E1) or return to pathway. We never observed phloem contact transitioning directly to non-probing. Phloem salivation can transition either to phloem ingestion (E2), or to pathway. Phloem ingestion could transition back to phloem salivation, or return to pathway. These results visually demonstrate the degree of plasticity in the consummation of feeding behaviors: ingesting either from phloem or xylem. Even with this non-statistical analysis, one sees a pattern wherein differences in leaf age are more obvious than differences between adaxial and abaxial (Objective 1), and xylem ingestion is a common part of *D. citri* behavior (Objective 2).

### Univariate Analysis: ANOVA

Forty-one out of the 85 variables tested showed significant differences among treatments (Table S-1 in Supplemental Information). All 41 variables showed some difference between psyllids on immature versus mature leaves, but only 14 variables indicated differences in psyllid feeding between abaxial and adaxial leaf surfaces. Of these 14 variables, eight variables clearly distinguished abaxial versus adaxial on immature leaves and four other variables did so for older leaves. All of these variables were associated with E1 and E2. However, there were 8 cases where the adaxial treatment on immature leaves was not significantly different but intermediate between abaxial surface on immature leaves and the outcome on mature leaves. This intermediate state suggests trends among some variables that support slight differences in psyllid behavior between abaxial versus adaxial surface.

Table 1 shows the findings for the 17 most highly significant variables among the 41 significant outcomes. Twelve of these 17 significant variables involved phloem salivation and phloem ingestion. The following interpretation of the values for these variables (Table 1) supports that psyllids altered their phloem activities depending primarily upon leaf age, and secondarily upon which side of the leaf they were feeding.

**Table 1:** Raw Means ± standard deviations, and LSD test for significant differences among EPG variables associated with *D. citri* ingestion behaviors on different leaf surfaces

Variable	Immature leaf	Immature leaf	Mature leaf	Mature leaf	F	Pr > F
	Adaxial	Abaxial	Adaxial	Abaxial		
PrcntPrbD	0.24 ± 0.21 B	0.40 ± 0.42 B	0.59 ± 1.07 A	0.45 ± 0.73 A	9.6	<0.0001
DurFirstE	5598.41 ± 8653.60 A	5656.39 ± 8108.44 A	30.21 ± 76.53 B	24.79 ± 72.37 B	13.54	<0.0001
CntrbE1toE	12.16 ± 31.21 C	2.67 ± 3.65 C	21.50 ± 38.48 A	7.51 ± 22.36 B	19.19	<0.0001
NumE1	1.58 ± 1.98 B	3.81 ± 3.89 A	1.00 ± 2.31 B	1.36 ± 3.28 B	6.5	0.0005
TtlDurE2	10,034.20 ± 12,352.84 A	15,622.47 ± 16,697.26 A	72.86 ± 266.62 B	1436.92 ± 4177.67 B	14.96	<0.0001
MnDurE2	12,318.07 ± 8710.79 A	8010.10 ± 5572.60 A	267.39 ± 273.15 C	4754.33 ± 4229.11 B	16.83	0.0001
NumE2	0.95 ± 1.22 B	2.24 ± 2.24 A	0.21 ± 0.54 C	0.52 ± 1.45 BC	10.98	<0.0001
DurMxE2	14,050.11 ± 8200.67 A	12,515.71 ± 8190.62 A	443.57 ± 578.03 B	7604.88 ± 6954.84 A	17.11	<0.0001
NumLngE2	0.89 ± 1.10 B	1.89 ± 1.85 A	0.05 ± 0.23 C	0.20 ± 0.58 C	16.47	<0.0001
PrcntPrbE2	18.51 ± 22.80 B	32.01 ± 27.99 A	0.16 ± 0.55 C	3.04 ± 8.94 C	18.78	<0.0001
TtlDurE1FllwdE2PlsE2	10,186.84 ± 12,547.97 A	15,913.35 ± 16,933.56 A	141.27 ± 406.02 B	1629.80 ± 4597.68 B	14.41	<0.0001
TtlDurE	10,247.34 ± 12,567.96 A	16,309.99 ± 17,683.83 A	198.48 ± 545.84 B	1681.56 ± 4636.37 B	14.19	<0.0001
TmFrstSusE2StrtPrb	1800.30 ± 1717.43 B	1808.21 ± 2074.54 B	3933.28 ± NA	16,022.29 ± 20,038.31 A	7.68	0.0004
TtlDurG	4055.84 ± 2749.78 B	3761.84 ± 4834.41 B	13,237.37 ± 10,663.17 A	12,559.85 ± 11,157.25 A	10.63	<0.0001
MnDurG	1864.46 ± 1309.90 B	1844.79 ± 1862.65 B	5652.08 ± 5248.06 A	5578.25 ± 6390.71 A	6.83	0.0003
PrcntPrbG	12.08 ± 10.92 B	14.20 ± 19.31 B	43.23 ± 27.73 A	32.66 ± 22.91 A	10.51	<0.0001
SdG	622.87 ± 376.85 B	449.09 ± 408.59 B	5469.52 ± 6262.26 A	3027.05 ± 2105.53 A	8.28	0.0003

All numbers (means, total duration, counts) are the expected values of the listed by insect variable. Mean comparisons are only valid within rows. Pr > F is the probability of a greater F value under the null hypothesis for the model. In all cases the numerator degrees of freedom = 3 and the denominator degrees of freedom = 96. Units are given in the footnote, but variables that are percentages or ratios are unitless

*In alphabetical order, the acronyms are: CntrbE1toE) Contribution of E1 to E; DurFirstE) Duration of first E1 (s); DurMxE2) Duration of longest E2 in recording (s); MnDurE2) Mean duration of E2 (s); MnDurG) Mean duration of G (s); NumE1) Number of E1 (count); NumE2) Number of E2 (count); NumLngE2) Number of long E2 (count); PrcntPrbD) Percent probe in D; PrcntPrbE2) Percent probe in E2; PrcntPrbG) Percent probe in G; SdG) Standard deviation of the Mean Duration of G (s); TmFrstSusE2StrtPrb) Time to first sustained E2 from start of probe with first sustained E2 (s); TtlDurE) Total Duration of E1 + E2 (s); TtlDurE1FllwdE2PlsE2) Total duration of all E1 followed by E2 plus duration of all E2 (s); TtlDurE2) Total duration E2 (s); TtlDurG) Total Duration of G (s)

Percentage of a probe in phloem contact (PrcntPrbD; Table 1) was significantly shorter on immature leaves, but there was no effect due to abaxial versus adaxial surface. The duration of the first E (DurFirstE; which includes both E1 and E2 when E2 follows the first E1) was longer for psyllids on immature leaves. Thus, when a psyllid on an immature leaf encountered the phloem for the first time it was more likely to stay there than to leave and begin seeking another vascular cell or withdraw from the plant. Out of 55 insects from all treatments with at least one E, 23.6% did not have either a G or E thereafter, 61.8% returned to E, and 12.7% switched to G. Overall, E1 contributes less to E (CntrbE1toE) on either surface of immature leaves than on either surface of mature leaves. Furthermore, while E1 contributed more to E on the adaxial surface of mature leaves than on the abaxial surface, this relationship was not significant on immature leaves. In addition, the number of E1 events (NumE1) was significantly greater on the abaxial surface of immature leaves relative to all other treatments (Table 1). Thus, there are more attempts to achieve phloem ingestion on immature leaves, but the relative contributions of D and E1 to phloem activities decline as a part of the D-E1-E2 pattern.

Psyllids spent more time in phloem ingestion per insect (i.e., total duration of E2, TtlDurE2, Table 1) on immature leaves versus older leaves, with no difference between surfaces on each leaf type. This finding for per-insect E2 durations was caused by a combination of per-event E2 durations and numbers of E2 events. When all E2 event durations were averaged (mean duration of E2, MnDurE2), the psyllid ingested for longer per event on immature leaves than on mature leaves, with no difference between abaxial and adaxial surface of immature leaves but a significant difference on mature leaves. The number of E2 events (NumE2) was greatest on the abaxial surface of immature leaves. There were significantly fewer on the adaxial surface of immature leaves, and fewer still on adaxial surface of mature leaves.

Most of the other variables associated with phloem activities (D, E1, or E2) in Table 1 (i.e., duration of the longest E2 event in a recording, DurMxE2; number of long E2 events, NumLngE2; percentage of probing represented by E2, PrcntPrE2; total duration of E1 followed by E2 plus duration of all E2 events, TtlDurE1FllwdE2PlsE2; and total duration of E [per insect], TtlDurE) showed larger values (on average 10.8 times larger, standard error 2.3) for psyllids in phloem of immature leaves versus mature leaves. A few variables also showed differences between abaxial and adaxial surfaces with larger values on the abaxial surface. For example, DurMxE2 had a significant difference between abaxial and adaxial for mature leaves (94% less on adaxial surface), while NumLngE2 and PrcntPrbE2 showed a significant difference between abaxial and adaxial surface of immature leaves (average 47.5% less on adaxial, standard error 0.053). Lastly, time to the first sustained E2 event from the start of probing (TmFrstSusE2StrtPrb), was shorter on immature than mature leaves (83% shorter), demonstrating that psyllids found the phloem faster on immature leaves.

Many of these differences could only be detected because we used a longer recording time than for most EPG studies. For example, average time to first phloem ingestion (E2) was 11 h on the abaxial side of an immature leaf, and 17 h on the abaxial side of a mature leaf. For psyllids feeding on the abaxial side of immature leaves, eight insects did not reach E2, and 29 did reach E2, with a total of 147 E2 events in a 22 h recording. In contrast, if recordings were limited to 8 h (a more typical duration), then recordings of 30 out of 37 psyllids would have lacked consummatory E2 behavior, and only 17 E2 events would have been performed by the remaining seven psyllids. A total of 32 phloem ingestion events in 19 insects were recorded on the adaxial side of an immature leaf in 22 h; eight of 19 insects failed to reach E2. The shortest time to E2 in these insects was 9.8 h.

In summary, our phloem findings show that psyllids choose to ingest phloem on abaxial surfaces of immature leaves, and avoid this behavior on adaxial surfaces of mature leaves. Behavior on the adaxial surface of immature leaves is more similar to behavior on the abaxial surface of immature leaves than it is to behavior on the abaxial surface of mature leaves; often the difference between the abaxial and adaxial surface of immature leaves was not significant.

Variables associated with xylem ingestion (G) showed a clear separation between mature leaves and immature leaves (Table 1). The total duration of G (TtlDurG, Table 1), the mean duration of G (MnDurG), the percentage of the probe in G (PrcntPrbG), and the standard deviation for the mean duration of G (SdG) were all significantly shorter for psyllids on immature leaves compared with psyllids on mature leaves. None of these variables showed an effect of abaxial versus adaxial leaf surface. In addition, we tested to see how often psyllids ingested xylem fluids after performing at least one event of phloem ingestion (E2). We found that 20–50% of the psyllids performed G after the first E2 event (Table 2). On immature abaxial surfaces, 47.9% of the 23 psyllids with D had a least one G event after the first D event; on mature adaxial leaves, the percentage increased to 85.7%.

**Table 2:** Probability of xylem ingestion (G) once *D. citri* has contacted (D), salivated into (E1), or ingested from (E2) phloem sieve elements

	D		E1		E2	
	N	%	N	%	N	%
Immature adaxial	13	30.8	11	27.3	9	22.2
Immature abaxial	23	47.9	21	42.9	21	42.9
Mature adaxial	7	85.7	5	60.0	3	33.3
Mature abaxial	10	60.0	5	60.0	4	50.0

N is the number of insects in the treatment with the behavior at the top of the column, while % is the percentage that ingested from xylem after that behavior

Overall, most variables were different between immature and mature leaves, and a few showed significant differences between abaxial and adaxial surface. On immature leaves, xylem ingestion was shorter per insect because G events were less common and of shorter duration on immature leaves compared with mature. Behaviors associated with phloem contact, phloem salivation, and phloem ingestion were more common with longer durations on immature leaves. The effect of leaf surface was less pronounced and there may have been an interaction effect between age and surface.

### Multiple Regression Analysis

This analysis is a formal test with each EPG variable for the effect of leaf age and leaf surface. Out of 36 variables (for all activities, not just phloem and xylem) with a significant model, 31 showed a significant effect of leaf age, seven showed a significant difference of leaf surface, and eight showed a significant interaction effect (Supplement S-2). Because more variables showed a significant effect of leaf age relative to leaf surface, we concluded that the effects on phloem and xylem ingestion from leaf age were stronger than the effect from leaf surface or the interaction of these two factors.

### Multivariate Analyses

**Fig. 3:**
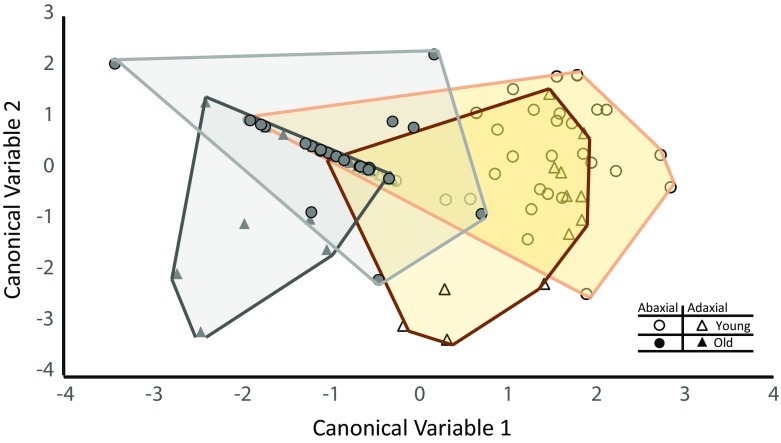
Plot of first two canonical variables derived from *D. citri* behavioral variables based on feeding on immature versus mature leaves and abaxial versus adaxial surface. This is a graphical representation of the range in behavior and the separation between the treatments

The entire data set was reduced to two canonical variables in the discriminant analysis (Table 3). Three canonical variables are listed, but the last one was not significant. Both of the significant canonical variables were functions of four univariate variables: 1) percentage of the total probing time spent in phloem contact (D), 2) percentage of total probing time spent in phloem ingestion (E2), 3) percentage of total probing time spent in pathway (C), and the 4) duration of the first E (E1 + E2). Slightly different mixtures of the four univariate variables occurred in each canonical variable. The result is Fig. 3. While there is considerable overlap in the four regions, the behavior of psyllids on older leaves has shifted the response area (gray plane) to the left (towards the y-axis) relative to psyllids on immature leaves (gold plane). Within each age group, feeding on the adaxial surface (triangles) has shifted the response area down (towards the x-axis) relative to psyllids on the abaxial surface (circles).

**Table 3:** Significance of the three canonical variables, the first two of which are plotted in Fig. 3

	Adjusted canonical correlation	Approximate standard error	Approximate F Value	Num DF	Den DF	Pr > F
1	0.631493	0.057514	6.39	12	246.35	<.0001
2	0.296697	0.088919	2.59	6	188	0.0197
3	0.186888	0.096926	1.75	2	95	0.1787

The canonical variables are a function of the percent of probe in D, percent of probe in E2, percent of probe in C, and duration of first E

The model had a significant Wilks lambda (F = 6.39, df 12, 246, p > F < 0.0001), which was testing the null hypothesis that means of all classes are equal. The distance between the groups was significant in all cases except between psyllids feeding on the abaxial versus adaxial surfaces of mature leaves (Table 4). The influence of abaxial versus adaxial was not significant on mature leaves but was on immature leaves with a Mahalanobis distance of ~1. In contrast, the Mahalanobis distance for abaxial surface between immature and mature leaves was ~2.8, while the equivalent value for the adaxial surface was ~3.2. Thus, the effect of age was about three times greater than the effect of leaf surface on psyllid feeding behavior.

**Table 4:** Squared Mahalanobis distances in overall behavior of *D. citri* probing different citrus tissues

		Squared distance			Probability of a larger value		
		Immature	Immature	Mature	Immature		Mature
		Abaxial	Adaxial	Adaxial	Abaxial	Adaxial	Adaxial
Immature	Adaxial	1.00143			0.0209		
Mature	Adaxial	4.02612	3.26451		<0.0001	<0.0001	
Mature	Abaxial	2.80477	2.61647	0.48598	<0.0001	<0.0001	0.2872

### Histological Comparison of Sweet Orange Cultivars

**Fig. 4:**
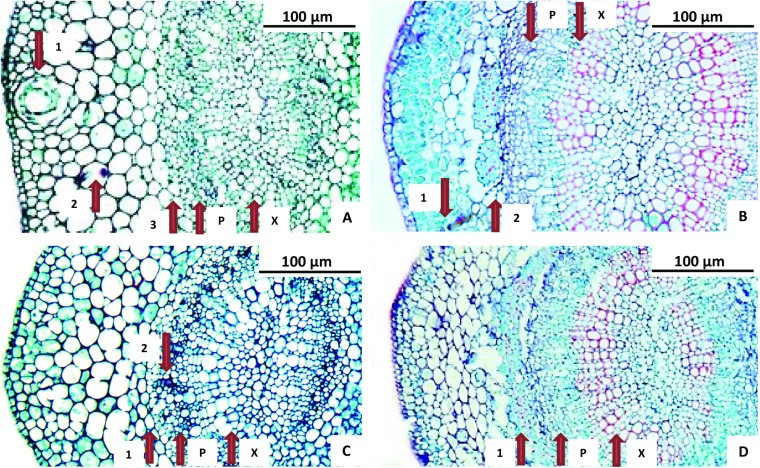
Histological comparison of immature and mature Midsweet and Valencia cultivar leaf midveins. The exterior abaxial surface of the midvein is to the left in each image. Note tissue tearing in both mature variety images, near the fibrous ring. The fibrous ring makes the leaf very tough and difficult to section. P = phloem. X = xylem. **a** Immature Midsweet. 1. Oil gland. 2. *D. citri* salivary deposits. 3. Location between parenchyma and functional phloem, showing absence of fibrous ring. **b** Mature Midsweet. 1. *D. ctiri* salivary deposits. 2. Fibrous ring- 3-5 cells thick. **c** Immature Valencia. 1. Location between parenchyma and functional phloem, showing absence of fibrous ring. 2. *D citri* salivary deposits. **d** Mature Valencia. 1. Fibrous ring- 3-5 cells thick

Our anatomical comparison of immature and mature Midsweet and Valencia sweet orange midveins showed no obvious varietal differences. Immature leaves in both varieties lack a fibrous ring (Fig. 4a, c), and both cultivars develop similar fibrous rings surrounding the vascular bundles as the leaves age (Fig. 4b, d), maturing to be 3–5 cells thick. Refer to Fig. 4 for more details.

## Discussion

### Are Psyllids Phloem Feeders or Xylem Feeders?

Some reports suggest that ingestion from xylem is uncommon in psyllids, which are thought to mostly ingest from phloem (Bonani et al. 2010; Zhu et al. 2010). We disagree with that conclusion. Our results conclusively showed that *D. citri* (uninfected with *C*Las) preferred to ingest from phloem sieve elements when they were on immature leaves and xylem cells when on mature leaves. Despite this pattern, it was also clear that psyllids on immature leaves occasionally chose to ingest from xylem, and psyllids on mature leaves occasionally ingested from phloem.

One of the goals of our study was to determine whether xylem ingestion was a natural part of the psyllid’s behavior or a stress response due to handling and wiring. If xylem ingestion were a handling stress response, then the insect would stop seeking xylem after it had discovered a path to phloem. We tested to see how often psyllids ingested xylem after ingesting phloem at least once. We found that a relatively high proportion (20–50%) of the psyllids ingested from xylem after the first phloem ingestion event. In addition, most psyllids on mature leaves that ingested from phloem would terminate that probe, then later return to ingest xylem. Along with the obvious shift in time spent ingesting xylem on mature leaves, these findings support the conclusion that xylem ingestion is a choice made by the psyllid. Thus, *D. citri* [like at least one leafhopper (Chuche et al. 2017)] is behaviorally plastic for ingestion choice, and to categorize the species as preferring phloem over xylem is overly simplistic.

Our evidence strongly supports that psyllids are facultative phloem- and xylem feeders, depending upon their position on the host plant. Studies have shown that choosing to ingest xylem is a natural behavior in aphids that is associated with general osmotic regulation, and therefore not just a stress response (Pompon et al. 2011; Spiller et al. 1990). Osmotic regulation may be important in psyllids, as in aphids, either because the psyllid needs water for osmoregulation or because it does not need additional nutrition for reproduction or dispersal. This conclusion is based on an observation that xylem in citrus was little more than a 5× dilution of phloem (Hijaz and Killiny 2014; Killiny and Hijaz 2015). Furthermore, reproduction is only possible on new flush, and new flush is not present during winter months. During this time, psyllids are found on mature leaves but would only need enough nutrition and water to survive until flush was once again available. Xylem ingestion may satisfy these requirements more easily than phloem.

In addition to the importance of chemistry for tissue selection, recent histological evidence (Ammar et al. 2013, 2014) has shown that constitutive anatomical defenses like thicker fibrous rings (sclerenchyma, also called phloem fiber, or bast fiber cells) around phloem can influence psyllid behavior. The differences in abaxial versus adaxial ingestion behavior may be partly attributable to morphological differences in the distribution of fibrous ring (Ammar et al. 2013). Additionally, the presence and thickness of the fibrous ring is determined in part by leaf age. In both Midsweet and Valencia cultivars of sweet orange, *Citrus sinensis*, the fibrous ring is absent in immature leaves (like those used in our study). However, the ringis present(but only one or two cell layers thick) in medium aged leaves (like those used in George et al. 2017), and deepens to 3 to 6 cell layers thick in mature leaves. These thickened cell walls of the fibrous ring present an anatomical barrier to psyllid probing that must be crossed or circumvented by the stylets to reach phloem or xylem. However, this would not explain why a psyllid that reached phloem would pull out of phloem and choose xylem ingestion some of the time. One would expect that physical barriers might result in differences in variables like time to first phloem ingestion (E2), while having little or no effect in variables dealing with the proportion of time spent ingesting phloem versus xylem; however, this was not the case. We are left to conclude that anatomical defenses do not explain preference for xylem ingestion.

Recent literature documents many factors that could account for the observed behavioral differences in the psyllid based on leaf age and abaxial/adaxial position. In mature leaves, the midrib has a core of dead, lignified pith cells. Moving out from the pith, around this core are xylem cells, and then a layer of phloem, the cambial layer, the fibrous ring of sclerenchyma, parenchyma cells, and then the epidermis. The fibrous ring can have channels, and may have lateral gaps (Ammar et al. 2013). The thickness of the fibrous ring was implicated as a mechanical barrier in psyllid resistant plants (Ammar et al. 2014; George et al. 2017). While this structure does affect insect behavior, it does not explain our results because the psyllids must go through the phloem cells to get to xylem. Thus, the psyllid is already past the cuticle, parenchyma, and fibrous ring when it contacts the part of the phloem where ingestion may take place, and it must move past the phloem to reach the xylem. It is therefore unlikely that morphological differences will determine whether psyllid adults will select phloem or xylem.

Many other factors could account for the observed behavioral plasticity of psyllids, based on leaf age and abaxial/adaxial position. Although it is not in the scope of this paper to thoroughly review recent research, such factors could include: 1) presence or structure of wax on mature versus immature leaves, 2) the role of chemical defenses such as compounds in non-vascular cells along the pathway, or translocated in phloem or xylem, 3) visual cues such as color to distinguish mature versus immature leaves, 4) various nutritional profiles of mature versus immature leaves, especially related to phloem versus xylem and the sink-source transition from immature to mature leaves (Turgeon 1989). Of these factors, nutrition may be especially important. The general conclusion of several recent papers (Hijaz et al. 2016; Killiny 2017; Setamou et al. 2016; Valim and Killiny 2017) is that the nutritional composition of phloem changes depending on the species, cultivar, and the age of the plant tissues. Based on this literature, we can conclude that there were nutritional differences between immature and mature leaves in our experiment, and similar experiments by others (George et al. 2017; Luo et al. 2015), as well as nutritional differences due to host species and cultivar used in each study.

Insects engage in compensatory feeding to overcome nutritional deficiencies in their diet (Couture et al. 2016). Based on the compensatory feeding hypothesis (Couture et al. 2016), one might expect that phloem ingestion on mature leaves would increase to compensate for reduced amino acid levels on mature leaves. However, this was not the case in our experiment. There are at least three possible reasons why this could happen. First, the mature leaves might have better chemical defenses. Second, these difference may be a strategic change in the psyllid wherein phloem ingestion is elevated on flush, in preparation for reproduction, while greatly reduced on mature leaves where the psyllid only needs to survive until flush is present. Third, the reduced nutritive content of phloem fluids in mature leaves could be a form of host plant resistance, as was demonstrated in a resistant sugarcane cultivar (Akbar et al. 2014; Hijaz et al. 2016).

### Comparison with Literature

Currently, there are two published articles that looked at the effect of leaf age on psyllid behavior (George et al. 2017; Luo et al. 2015). Both of these articles show some effect of leaf age on feeding behavior. However, the details of what behavioral changes take place are a bit different between these studies. We suggest that the results between these manuscripts and ours are not comparable because “young” or “immature” leaf is not a constant across studies and we have shown that leaves that are fully expanded but immature are morphologically distinct from leaves that are not fully expanded in that the immature leaves lack a fibrous ring. It is likely that our “immature leaf” is close to Luo’s “new shoot” category. The presence of any cell layers of fibrous ring will act as an impediment to *D. citri* probing behaviors performed in vascular tissues. The insects must either pass through or locate a gap in the phloem fiber cells in order to locate vascular tissues. The necessity of such searching delays the time to first access of vascular tissues and thusly the presence of an incompletely developed fibrous ring in immature but fully expanded leaves and a well-developed fibrous ring in mature leaves can be described as a resistance feature to *D. citri* probing.

Also, EPG researchers should take note from this study of the importance of a long recording time for psyllids. An 8 h recording time (standard for aphid recordings) is not appropriate for these insects. Especially in treatments where it took psyllids much longer than 8 h to reach phloem, incorrect conclusions would be reached with a short recording time. For example, had we used an 8 h recording time, we would have concluded that the psyllid was incapable of feeding on the adaxial surface of leaves.

### Implications for Insecticide Studies

How much time does it take an insecticide to kill a psyllid? Is it enough time to prevent either acquisition or inoculation of *C*Las by *D. citri*? Both parts of transmission occur when stylets reach the phloem. Therefore, the answer depends upon leaf surface and age used for the test. Our results showed that psyllids reach the phloem more quickly (in about 7.5 versus 12.5 h, on average) and reliably (75% of insects versus 38% on average) when they feed while standing on the abaxial surface of immature leaves. This range is consistent with previously published results (Grafton-Cardwell et al. 2013; Inoue et al. 2009; Pelz-Stelinski et al. 2010). Therefore, insecticide experiments should use the abaxial surface of immature leaves for assessing insecticide efficacy. The minimum duration before phloem ingestion on abaxial immature leaves was only 8.4 min, despite the average being many hours. Thus a few insects reach phloem quickly, and the important issue involves deciding what level of insecticide protection is sufficient and if it is possible, legal, or economical to apply sufficient insecticide to achieve that goal.

### Conclusions

We have documented that *D. citri* not infected with *C*Las choose (most of the time) to ingest phloem on immature citrus leaves, while those on mature leaves choose xylem. During winter, when flush is scarce, this may result in a reduced rate at which an uninfected adult psyllid can acquire *C*Las. The rate at which an infected adult inoculates may also be reduced in times of minimal flush.

We asked four questions in the introduction to this manuscript that we can rephrase as statements given the results from our study. 1) The strength of the effect of leaf age is greater than the effect of feeding location (abaxial/adaxial). However, there is a statistically significant difference between feeding on the abaxial versus adaxial leaf surface. 2) *D. citri* is predominantly a phloem feeder on immature leaves, and predominantly a xylem feeder on mature leaves of favorable hosts. 3) An insecticide must act within a few minutes to guarantee protection because our minimum observed time to first E2 (phloem ingestion) was 8.4 min.

The primary method for managing Huanglongbing is by controlling the vector using insecticides. Insecticide research focused on showing that an insecticide prevents the psyllids from contacting the phloem should focus experiments on psyllids that are given access to immature leaves where there is a better chance that the psyllid will attempt and succeed in ingesting from phloem.

## Electronic supplementary material

ESM 1 (XLSX 34 kb)
